# Atomic Fabrication of 2D Materials Using Electron Beams Inside an Electron Microscope

**DOI:** 10.3390/nano14211718

**Published:** 2024-10-28

**Authors:** Mingrui Zhou, Wei Zhang, Jinyi Sun, Fuqiang Chu, Guocai Dong, Meng Nie, Tao Xu, Litao Sun

**Affiliations:** 1 SEU-FEI Nano-Pico Center, Key Laboratory of MEMS of Ministry of Education, Southeast University, Nanjing 210096, China; 2 Jiangnan Graphene Research Institute, Changzhou 213149, China

**Keywords:** atomic fabrication, electron beam irradiation, two-dimensional materials, transmission electron microscope, scanning transmission electron microscope

## Abstract

Two-dimensional (2D) materials have garnered increasing attention due to their unusual properties and significant potential applications in electronic devices. However, the performance of these devices is closely related to the atomic structure of the material, which can be influenced through manipulation and fabrication at the atomic scale. Transmission electron microscopes (TEMs) and scanning TEMs (STEMs) provide an attractive platform for investigating atomic fabrication due to their ability to trigger and monitor structural evolution at the atomic scale using electron beams. Furthermore, the accuracy and consistency of atomic fabrication can be enhanced with an automated approach. In this paper, we briefly introduce the effect of electron beam irradiation and then discuss the atomic structure evolution that it can induced. Subsequently, the use of electron beams for achieving desired structures and patterns in a controllable manner is reviewed. Finally, the challenges and opportunities of atomic fabrication on 2D materials inside an electron microscope are discussed.

## 1. Introduction

Over the past few decades, significant progress has been made in the effort to scale existing fabrication technology to smaller feature dimensions. However, it is anticipated that fabrication capabilities are approaching their limits [1,2,3]. 2D-material semiconductors, such as transition metal dichalcogenides (TMDs), phosphorene, etc., have gained increasing interest as atomically thin channels that could facilitate continued transistor scaling [3]. Furthermore, some structures derived from 2D materials, such as graphene nanoribbons, exhibit significant advantages [4] and are promising candidates as the building blocks for next-generation electronic devices. However, the fabrication of atomically tailored structures from 2D materials remains an outstanding challenge.

Atomic fabrication is not a new concept, being first proposed by Feynman in 1959 [5] and then elaborated by Drexler in his book *Engines of Creation* [6]. Since the invention and implementation of the scanning probe microscope (SPM), a series of studies have been carried out in order to manipulate matter at the atomic level [7,8]. However, manipulation based on an SPM requires harsh conditions [9], such as ultra-low temperatures and atomically clean surfaces [10,11].

The transmission electron microscope (TEM) and scanning TEM (STEM, either as a dedicated microscope or as an operation mode integrated into a TEM) have attracted increasing attention because of their capability to both characterize a specimen with atomic resolution and drive material alterations at the atomic scale [12,13,14,15,16]. The focused electron beam in a TEM or STEM can be used to directly pattern the specimen based on the interaction between electrons and matter [17,18]. Furthermore, environmental stimuli can be introduced into a (S)TEM by modifications to the microscope or the development of dedicated specimen holders to achieve atomic fabrication under realistic conditions [9].

To date, many efforts have been made to fabricate nanostructures inside the (S)TEM manually. However, in some cases, the fabrication cannot meet a high precision, high efficiency, and high consistency. Automation methods, particularly based on machine learning, are expected to solve this issue. Sample information regarding the local structure (atom coordinates [19,20], image contour [21], defect structures [22,23], etc.) can be collected and analyzed in real time, providing the basis for a rapid adjustment of the fabrication strategy and controlling parameters that guide the fabrication process toward the desired structure.

In this paper, we concentrate on the atomic fabrication of 2D materials using electron beam irradiation inside a (S)TEM. Firstly, the effect of electron beam irradiation is briefly introduced, followed by a discussion on the control of electron beam irradiation damage. Subsequently, we discuss the various structural dynamics observed in 2D materials arising due to electron beam irradiation, as well as examples of the manipulations and fabrications of 2D structures made possible through these effects. Finally, we discuss the challenges for further developments in atomic fabrication using (S)TEMs.

## 2. Fundamental Mechanism of Electron Beam Irradiation

**Figure 1:**
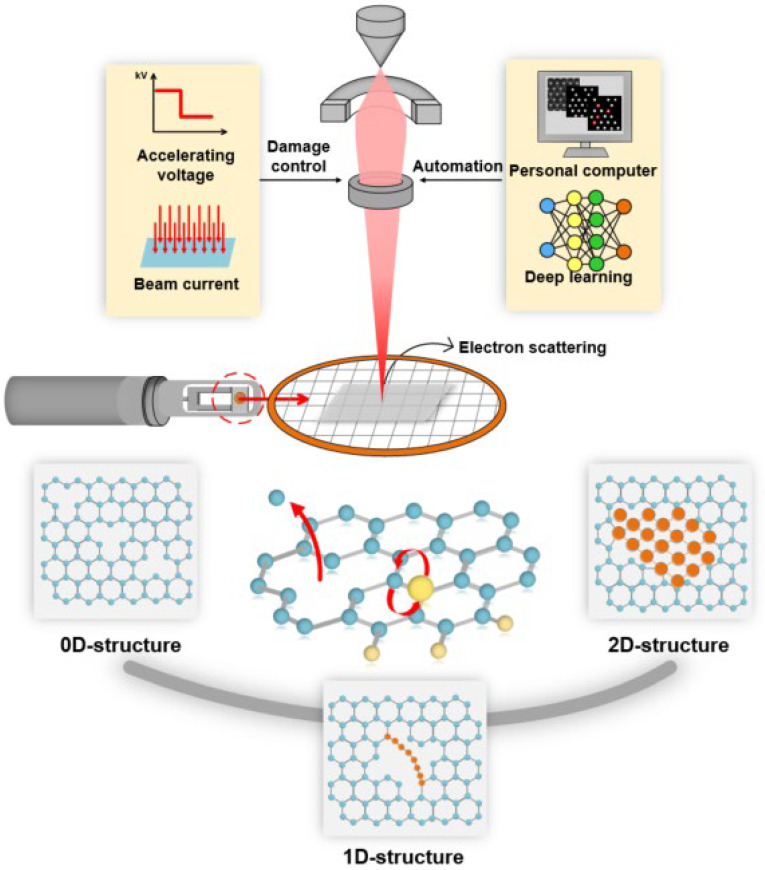
Schematic illustration of atomic fabrication using an electron beam inside a (S)TEM. The interaction of electrons with the specimen can trigger the structural evolution of the specimen, which is the basis of atomic fabrication and can be controlled by the manual or automatic adjustment of electron beam parameters.

When the specimen is exposed to energetic electron bombardment, either elastic or inelastic collisions between electrons and the specimen can cause temporary or permanent changes in the structure and chemistry of the specimen. As shown in Figure 1, appropriate irradiation can promote the structural evolution of the specimen, which is a fundamental process for atomic fabrication. Fully understanding the effects and the influence mechanism of electron beam irradiation represents a significant step toward achieving the goal of atomic fabrication.

### 2.1. Elastic and Inelastic Scattering

Inelastic scattering represents the interaction of incoming electrons with the atomic electrons surrounding the nucleus, which gives rise to several effects, mainly including radiolysis, heating, and electrostatic charging [24]. Radiolysis is the ionization of atoms or breaking of chemical bonds, which is serious in insulators. Electron-beam-induced heating results from the energy transferred from incoming electrons to the atomic lattice, manifesting as an increase in sample temperature. This effect is usually negligible except for specimens with relatively low thermal conductivity. Electrostatic charging occurs primarily in electrically insulating specimens. These effects may increase the instability of the material, leading to structural changes and even mass loss [25].

Elastic scattering occurs when incident electrons collide with the atomic nucleus without losing energy. It can result in an atomic displacement within crystalline specimens and the sputtering of atoms from a specimen’s surface. A displacement occurs when the transferred energy from incoming electrons is large enough that the nucleus is knocked to an interstitial position, resulting in a degradation of the crystalline perfection.

Taking into account energy and momentum conservation, the energy transferred to the nucleus can be expressed as follows:

(1) $$E=E_{max}{sin}^{2}(\frac{\theta }{2}),$$

where *θ* is the scattering angle, and *E*_max_ is the maximum transferred energy corresponding to a head-on collision with a scattering angle of 180°, which is a function of the incident-electron energy *E*_0_:

(2) $$E_{max}=\frac{2E_{0}(E_{0}+2{m_{e}c}^{2})}{Mc^{2}},$$

where *m_e_* is the mass of the electron, *M* is the mass of the nucleus, and *c* is the speed of light.

Atoms can be knocked away from the lattice site when *E* exceeds the displacement energy *E_d_* (an intrinsic parameter of the material related to the bond strength, crystal lattice, and atomic weight of the constituent atoms) [26]. Correspondingly, atomic displacement only occurs when the energy of the incident electron is larger than the threshold energy *E_th_* (the value of *E*_0_ in Equation (2) in the case of *E*_max_ = *E*_d_).

Atoms are free to leave the specimen if high-angle elastic scattering occurs at the surface, which is known as sputtering [27].

Atomic sputtering primarily occurs on the beam-exit surface when the transferred energy is larger than the sputtering energy *E_s_*. *E_s_* is much lower than *E_d_* in the bulk because surface atoms are always less tightly bound than bulk atoms. *E_s_* is often in the range of 1~2 *E_sub_*, while *E_d_* is in the range of 4~5 *E_sub_*, where *E_sub_* is the sublimation energy [25,28]. Notably, some sputtered atoms remain adsorbed and weakly bound to the sample surface. These atoms tend to diffuse easily and provide a source for surface growth and reconstruction.

### 2.2. Damage Control

It is well known that electron beam irradiation damage is related to electron beam parameters, wherein the energy of the incident electron (accelerating voltage) and electron dose/dose rate are frequently used to control electron irradiation.

Both elastic and inelastic scattering effects are closely related to the incident electron energy. An optimum accelerating voltage should be chosen depending on the materials under study. For conductive 2D materials, such as graphene, electron irradiation damage is primarily driven by elastic scattering [29]. Consequently, an incident energy larger than *E_th_* is favorable for subtractive fabrication with atom loss via displacement and sputtering [30,31,32], which increases with an increasing electron energy in the range of 1~2 *E_th_*. Although the loss of atoms can be avoided when the incident energy is lower than *E_th_*, the materials and structures can also be tailored via bond rotation, vacancy reconstruction, and chemical reaction [33]. For 2D semiconductors and insulators, inelastic scattering effects should be taken into account and even can be predominant, especially when the incident energy is below *E_th_*. Generally, the damage caused by inelastic scattering increases with decreasing electron energy within limits. For example, the damage on 2D WS_2_ under a 30 keV electron beam is twice that under a 60 keV electron beam [33]. As a result, finding a voltage value that balances the effects of elastic and inelastic scattering is crucial for controlling radiation damage in 2D semiconductor materials.

On the other hand, the amount of radiation damage is generally proportional to the total electron dose. Therefore, dose rate (electron beam current density) and exposure time are the two aspects primarily used to control damage. For instance, beam current density increases as the electron beam is focused. A beam focused onto a spot smaller than 0.1 nm can reach a current density of 10^7^ A/cm^2^ and be used for material sculpting. It should be noted that irradiation damage is only observed when the dose rate exceeds a critical value for certain materials; thus, a sufficient electron beam current density is essential for further fabrication.

## 3. Atomic Dynamics of 2D Materials Under Electron Beam Irradiation

The structural evolution of a specimen is the essence of atomic fabrication. Electron beam irradiation may cause atom loss, atom rearrangement, and even atom gain. Meanwhile, these events can be monitored and distinguished using the same electron microscope. Knowledge of the atomic arrangement and evolution allows for an examination and exploration of the factors that influence the dynamic changes brought on by the electron beam. Gaining insight into these effects is crucial for the controlled manipulation of 2D materials at the atomic scale.

### 3.1. Atomic Etching

**Figure 2:**
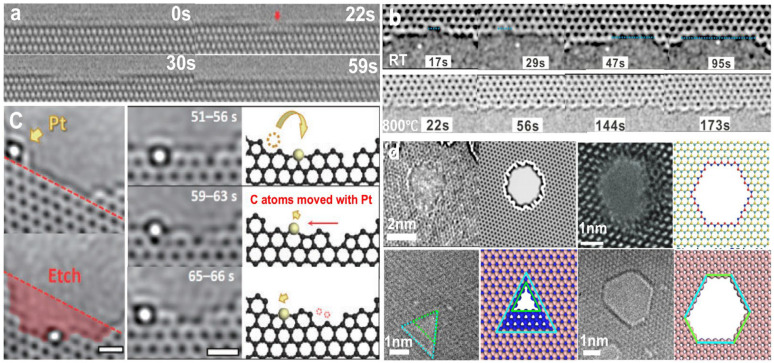
Atomic etching under electron beam irradiation. (**a**) TEM image sequence showing row-by-row etching along the zigzag edge in monolayer MoS_2_. Reprinted with permission from Ref. [37]. Copyright 2017, American Chemical Society. (**b**) Temperature-dependent etching. Reprinted with permission from Ref. [15]. Copyright 2015, American Chemical Society. (**c**) Pt atom-assisted etching. Scale bar: 0.5 nm. Reprinted with permission from Ref. [42]. Copyright 2017, The Japan Society of Applied Physics. (**d**) Structure-dependent etching. Reprinted with permission from Ref. [43]. Copyright 2012, PNAS. Reprinted with permission from Ref. [44]. Copyright 2018, WILEY-VCH Verlag GmbH & Co. KGaA, Weinheim, Germany. Reprinted with permission from Ref. [45]. Copyright 2022, The Royal Society of Chemistry.

Atomic etching, particularly at the edge or on the surface of a material, can result from either elastic or inelastic scattering and has been widely investigated in 2D materials using (S)TEMs [34,35,36]. Figure 2a presents a typical example of etching along the zigzag edge in monolayer MoS_2_ using a 60 keV electron beam [37]. The etching typically starts at a single point and propagates in both directions along the edge until the entire atomic row is removed. During the etching, the edge S atoms are preferentially ejected due to their higher sputtering cross-section, leading to bonding between rows of Mo atoms. The electron beam lacks sufficient energy to sputter Mo atoms but can activate edge migration, leaving Mo-terminated zigzag edges. A similar etching process has also been observed in graphene [38], h-BN [39], and some other layered materials [40,41].

Thermal effects have been demonstrated to influence electron beam etching [46,47,48]. As shown in Figure 2b, the etching rate of C atoms under 60 keV electron beams at elevated temperatures is slower than at room temperature (RT). Because the incident electron energy is below the displacement threshold, the rapid sputtering rate at low temperature is dominated by the chemical reaction between graphene and surface contaminations. As the temperature increases to above 600 °C, the contamination evaporates off and then the chemical etching diminishes dramatically [46,47,48]. Meanwhile, the annealing of structural defects with mobile carbon adatoms can further slow sputtering [49,50]. Therefore, etching at high temperature is slower. This observation challenges the conventional notion that a higher energy level is directly correlated with an increased etching rate, offering a deeper understanding of the irradiation process. It is noted that the edge terminations are also temperature-dependent. Below 400 °C, the etched edges of graphene mainly exhibit zigzag terminations; while above 600 °C, armchair and reconstructed zigzag edges are predominant due to higher thermodynamic stability [46,47,48]. Another example is that only N-terminated zigzag edges are frequently formed under 80 keV electron beam irradiation at low temperatures, while both N- and B-terminated zigzag edges become prominent at temperatures above 700 °C [51]. At low temperatures, surface residue can preferentially remove B atoms, leaving exclusively N-terminated edges. At high temperatures, the removal of residue from the specimen eliminates the asymmetry in the chemical reactivity of B and N. Therefore, B and N may be ejected with comparable probability, resulting in both N- and B-terminated edges.

Foreign atoms may also assist in etching. Taking graphene as an example, some metal atoms (such as Pt [42], Au [52], Sn [53], etc.) have been confirmed to assist in edge etching. As shown in Figure 2c, the Pt atom moves into a vacancy and the nearby C atoms are displaced due to the size mismatch between the Pt atom and vacancy. The Pt atom is less mobile and strains the edge, which reduces the knock-on threshold of local C atoms, thus promoting etching. In addition, some nonmetal atoms, such as Si [54], can also assist in the etching of graphene. Conversely, catalyst-assisted etching can also be tuned by electron irradiation and other external stimuli, representing a new paradigm to fabricate stable nanostructures with high precision.

Etching behaviors are also related to the material of the specimen. Because irradiation damage is element-dependent and structure-dependent, structural evolution and as-formed structures are distinct in different 2D systems. As shown in Figure 2d, the nanopores formed at RT are circular in graphene [43], hexagonal with Mo- and S-terminated zigzag edges in MoS_2_ [44,55], and triangular with N-terminated zigzag edges in h-BN [45]. For bilayer or few-layer 2D materials, the stacking sequence also affects etching behavior. For example, the shape of as-formed nanopores in AA′- and AB-stacked bilayer h-BN is distinct [45]. When AA’-stacked h-BN is irradiated by an electron beam, the triangular nanopores formed in different layers have opposite orientations and overlap to form a hexagonal pore. In the case of the AB-stacked bilayer, the triangular nanopores in different layers have the same orientations, and overlapped pores in the bilayer maintain a triangular shape.

### 3.2. Atomic Growth

The extraordinary performance of 2D materials is strongly dependent on the growth conditions, yet the growth mechanism is not fully understood. The use of an in situ TEM enables dynamical observation at the atomic scale and an investigation of the influencing factors on atomic growth.

**Figure 3:**
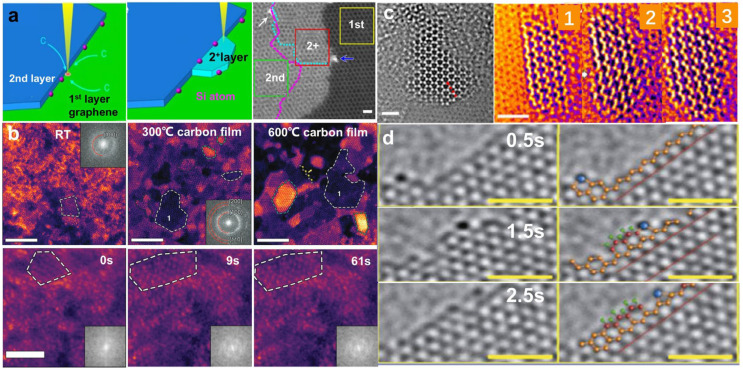
Atomic growth caused by electron beam irradiation. (**a**) In-plane growth of graphene at the edge. Scale bar: 0.5 nm. Reprinted with permission from Ref. [58]. Copyright 2014, Macmillan Publishers Limited. (**b**) Thermal-assisted formation of MoS_2_ flakes at RT, 300 °C, and 600 °C. Scale bar: 5 nm. The bottom is the growth of MoS_2_ from an amorphous precursor at RT. Scale bar: 2 nm. Reprinted with permission from Ref. [61]. Copyright 2019, WILEY-VCH Verlag GmbH & Co. KGaA, Weinheim. (**c**) Growth of ZnO on graphene. Scale bar: 1 nm. Reprinted with permission from Ref. [62]. Copyright 2017, American Chemical Society. (**d**) Cr atom-assisted growth of graphene. Scale bar: 1 nm. Reprinted with permission from Ref. [63]. Copyright 2018, Tsinghua University Press and Springer-Verlag GmbH Germany, part of Springer Nature.

The in situ growth of desired structures requires the introduction of appropriate precursors into the microscope. Residual hydrocarbon in the microscope chamber and adsorbed on the specimen can provide a carbon source for the growth of graphene [56,57]. Figure 3a presents an example of electron-beam-induced growth at the step-edge of a bilayer graphene substrate [58]. The electron beam plays a significant role in the growth, and thus, no growth is found in areas far from the scanned area. Such step-edge growth is only found in the second layer where another layer is necessary as the template for in-plane growth. However, the residue is usually slight, resulting in small-scale growth. The use of an environmental TEM, which continuously introduces precursor gas into the chamber, can effectively solve this issue. For instance, Liu et al. achieved larger-scale growth, both lateral epitaxial and vertical growth, under a CO_2_ atmosphere [59]. Significantly, such growth can be extended to other 2D materials [60].

Growth is also dependent on temperature. As shown in Figure 3a, in-plane graphene growth occurs only when the substrate graphene is heated to 500–700 °C, while the graphene is contaminated with amorphous carbon at RT [58]. Although the graphitization of amorphous carbon supported on flat sheets can be achieved under an electron beam, it is somewhat similar to high-temperature-induced crystallization [60]. Temperature-dependent transformation and growth are also observed in some other 2D materials [61]. Figure 3b presents the growth of MoS_2_ from a (NH_4_)_2_MoS_4_ precursor under 100 keV electron beam irradiation at various temperatures [61]. It has been found that MoS_2_ flakes grow larger, with more layers and clearer edge structures, as the thermolysis temperature increases. This can be attributed to the fact that high temperatures provide activation energy to atoms, making it easier to aggregate and rearrange them [61]. The growth of ordered nanograins at RT has also been found, which suggests that electron beams can provide additional energy for the formation of MoS_2_.

Substrates are essential for the growth of 2D structures. Börrnert et al. found that freestanding amorphous carbon preferentially converted into graphitic carbon onions, while amorphous carbon supported on flat 2D sheets transformed to a planar structure parallel to the substrate as a result of the van der Waals interaction between them [60]. Similarly, electron beams can trigger the crystallization of amorphous MoS_2_ (a-MoS_2_) on a graphene substrate, resulting in the formation of crystalline nanograins [64]. Furthermore, 2D substrates can provide a template for the epitaxial growth of novel structures. Figure 3c exhibits the in-plane growth of a ZnO monolayer from residual clusters or nanoparticles on a graphene substrate [60]. Zinc and oxygen atoms are observed to expand at the zigzag edge of the ZnO monolayer with a graphene-like structure. Interestingly, two dominant misorientation angles (0° and 30°) are associated with the epitaxial growth, and 0° becomes dominant as the ZnO grows [60]. In addition, the use of nanopores as growth templates is an extremely promising approach for the growth of freestanding (quasi) 2D metal/metal oxide membranes.

It is well known that catalysts play an important role in growth. Some metals, such as Fe, Cu, and Cr, are considered to be excellent catalysts in the growth of C materials [65]. Figure 3d presents the catalytic growth of a single Cr atom at the edge under 80 keV electron beam irradiation [64]. Compared with Fe, Cr is relatively stable and hence more efficient as a nucleation catalyst. The catalytic effects of Sn [53] have also been verified. Both growth and etching were observed when Sn atoms diffused along the graphene edges. These processes could be controlled by regulating the supply of C atoms.

### 3.3. Atomic Migration and Rearrangement

Point defects in 2D sheets, consisting of impurity atoms or vacancies, exhibit facile migrations under electron beam irradiation. Consequently, it becomes feasible to manipulate the motions of individual atoms or point defects and further fabricate complex structures [66].

**Figure 4:**
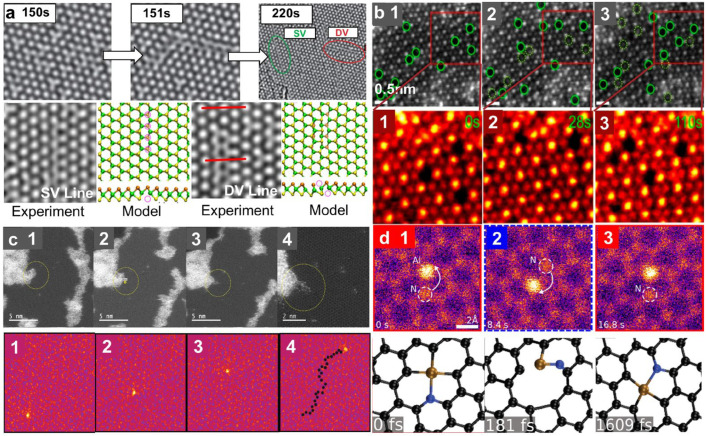
Atomic migration and rearrangement under electron beam irradiation. (**a**) Formation of line defects through the migration and aggregation of S vacancies in monolayer MoS_2_. Reprinted with permission from Ref. [67]. Copyright 2013, American Physical Society. (**b**) Migration of Se vacancies in monolayer PtSe_2_. Scale bar: 0.5 nm. Reprinted with permission from Ref. [71]. Copyright 2022, American Chemical Society. (**c**) Migration of Si atoms. Reprinted with permission from Ref. [10]. Copyright 2018, WILEY-VCH Verlag GmbH & Co. KGaA, Weinheim. (**d**) STEM image sequence and atomic model demonstrating the rotation of the Al–C_3_N site. Reprinted with permission from Ref. [33]. Copyright 2022, the author(s).

Taking 2H-phase MoS_2_ as an example, S vacancies are easy to form and migrate in a monolayer under electron beam irradiation. Further aggregation of vacancies may result in the formation of line defects, which has also been observed in other 2H-phase TMD sheets [11]. Figure 4a exhibits the formation of a single vacancy line and a double vacancy line in monolayer MoS_2_, where the two lines of vacancies are at the neighboring sites and opposite sides in a staggered configuration [67]. The orientation of line defects is sensitive to mechanical strain, thus the direction can be controlled by the introduction of an external strain [67]. The migration of S vacancies is more complex in bilayer MoS_2_, and both in-plane migration and interlayer migration have been observed [68]. Meanwhile, the structural deformation introduced by single vacancy lines is obvious in the monolayer but negligible in the bilayer system because of the competition between the van der Waals interlayer force and compression in the deformed layer [68]. In addition, thermal annealing could promote the migration and reconstruction of the defects, resulting in the formation of ultralong defects or complex structures [69]. Typically, the linear vacancies formed in TMD sheets at RT are a few nanometers in length, while the linear vacancies at high temperatures are atomically uniform over tens of nanometers [70].

The behavior of vacancies in 1T-phase TMDs exhibits distinct characteristics. In the case of PtSe_2_, Se vacancies (V_se_) have preferential sites with high beam-induced mobility and pair up into diverse divacancies rather than vacancy lines under 60 keV electron beam irradiation due to a higher probability of atomic loss compared to the diffusion required for creating vacancy lines [71]. Figure 4b shows the beam-induced movement of V_se_ in monolayer PtSe_2_ at 200 °C. The migration of V_Se_ involves the filling in of the original vacancies by other Se atoms from a pristine lattice and creating another V_Se_ simultaneously [71]. The motions of vacancies can also occur via bond rotations under electron beam irradiation [72,73,74].

The controlled migration of dopant atoms has also been successfully achieved in 2D materials [10,75]. Figure 4c exhibits the introduction of Si substitutional defects and defect clusters in graphene with the spatial control of a few nanometers using 100 keV electron beams and the controllable migration of individual Si atoms using a 60 keV electron beam [10]. The Si substitutional defects are further manipulated to form dimers, trimers, and more complex structures, which provide an enabling tool for atom-by-atom fabrication.

Meanwhile, the migration and rearrangement of some other foreign atoms triggered by electron beams have been observed in 2D sheets. Figure 4d shows the rotation of Al-N bonds in graphene driven by a 60 keV electron beam [33]. When a N atom is activated by the electron beam, it rotates over the Al atom while the Al atom fills the vacancy left by the N atom. Al and N atoms will return to their original positions under further irradiation, which means a non-destructive change is happening in this process.

## 4. Controllable Fabrication of 2D Materials Using Electron Beams

The fundamental goal of atomic fabrication is to manipulate atoms to obtain predefined structures with atomic precision. Inside the microscope, fabrication can be achieved by electron beam irradiation and, in some cases, through a combination with external stimuli. Point defects frequently appear in the primary period and gradually expand into complex structures or nanopores under electron beam irradiation. Furthermore, 1D and 2D structures can be achieved by moving electron beams along predefined paths.

### 4.1. 0D Structures

**Figure 5:**
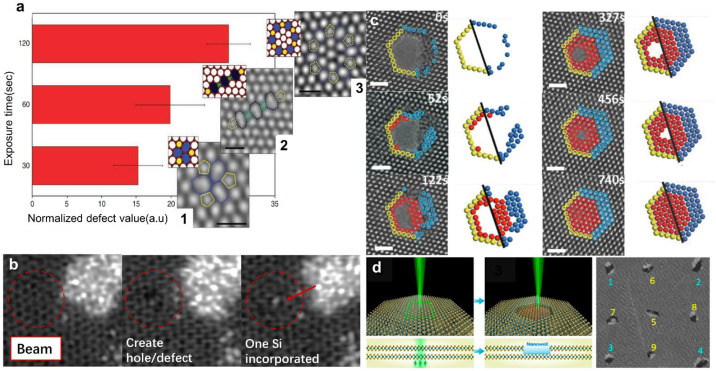
0D nanostructures. (**a**) Bar chart illustrating the relationship between exposure time and defect complexity. Scale bars: 0.5 nm. Reprinted with permission from Ref. [76]. Copyright 2012, Springer Nature Limited. (**b**) The introduction of a single Si substitutional defect.The region of interest is marked with a red circle. Reprinted with permission from Ref. [78]. Copyright 2017, AIP Publishing. (**c**) HRTEM images and atomic models showing the repair of MoS_2_ nanopores. Scale bar: 1 nm. Reprinted with permission from Ref. [44]. Copyright 2018, WILEY-VCH Verlag GmbH & Co. KGaA, Weinheim. (**d**) The fabrication of nanowells in bilayer WS_2_. Reprinted with permission from Ref. [79]. Copyright 2019, American Chemical Society.

Point defects and nanopores are two common types of 0D structures. In recent decades, focused electron beams inside (S)TEMs have been employed to fabricate 0D structures with controllable shapes and sizes by adjusting the beam parameters. Figure 5a shows the spatial control of defect creation in graphene with variable complexity by adjusting the exposure time [76]. The divacancy (DV) with various configurations is created via atomic sputtering under 80 keV electron beam irradiation with a beam current density of 10^3^ A/cm^2^. The isolated DVs can freely oscillate between three stable configurations via Stone–Wales rotations. Extending the exposure time will result in the joining of DVs and the formation of larger defect structures and nanopores. The creation and manipulation of point defects in some other 2D sheets have also been extensively studied [77]. For instance, point defects can be modified by bond rotations at high temperatures in W-based TMDs [77].

The formation of point defects can also be achieved by the introduction of dopant atoms into a specified location. Figure 5b shows the introduction of a single Si substitutional defect in graphene under electron beam irradiation [78]. The 100 keV electron beam is placed on the desired lattice site for 1~2 s, resulting in the formation of vacancies via atomic sputtering. Subsequently, the electron beam is moved to the Si/C source material for sputtering atoms into the graphene lattice, thus an individual vacancy is controllably passivated by Si substitutional atoms. Another impressive example is the precise doping of h-BN with C atoms [80].

Extending the exposure time may lead to the formation of nanopores. The pores sculpted by the focused electron beam usually exhibit an approximately circular shape with high symmetry in most instances but may evolve into a specific shape under mild parallel electron beam irradiation. The pore size may have a linear or exponential dependence on the dose when the electron energy is larger than the threshold energy [45]. Furthermore, both thermal treatment and electron beam irradiation can promote the migration of adatoms and the reconstruction of structural defects, which is expected to repair the as-sculpted nanopores [30,44]. Figure 5c shows the repair of nanopores in MoS_2_ sheets resulting in high-quality crystals with few defects under parallel electron beam irradiation with an energy of 300 keV and a beam current density of 10 A/cm^2^ [44]. The atoms are knocked away from the lattice by energetic electrons, thereby diffusing and rearranging at the edge of the nanopores to reduce the free surface energy. It should be noted that both the growth and repair of nanopores might happen, which is dependent on the beam current density [12].

Compared with vacancies and nanopores, the fabrication of nanowells is considered to be more challenging. Recently, Chen et al. employed an 80 keV focused electron beam to remove the atoms in one layer of bilayer WS_2_ at 800 °C and obtained various nanowells after ~40 s [79]. The schematic diagram and the image of nanowells are presented in Figure 5d. The authors also found that the formation of nanowells in the bilayer was different from the production of nanopores in the monolayer due to the van der Waals interactions between the adjacent layers.

### 4.2. 1D Structures Derived from 2D Sheets

Ultrathin 1D nanostructures, which may serve as interconnections for integrated circuits, have been fabricated using electron beams. Electron beam irradiation is an attractive approach for exploring material behavior at fundamental length scales, allowing the fabrication and examination of nanoribbons, wires, and atomic chains [81].

**Figure 6:**
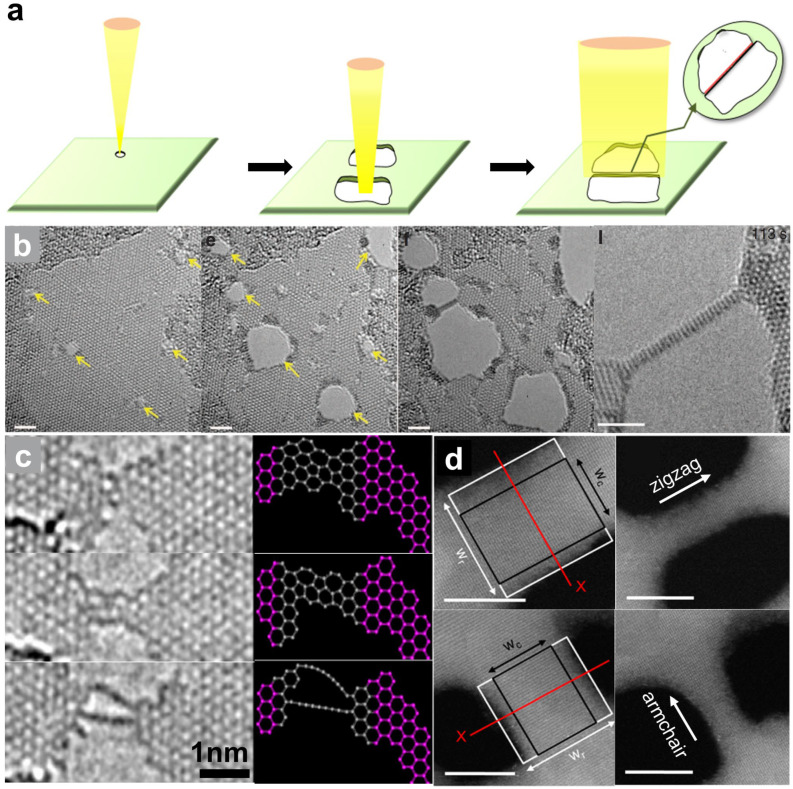
1D nanostructures. (**a**) The common strategy for the fabrication of 1D structures by electron beam irradiation. (**b**) The formation of molybdenum sulfide ribbons between two adjacent holes. Irradiation-induced holes are highlighted by the arrows. Scale bar: 2 nm. Reprinted with permission from Ref. [82]. Copyright 2013, the author(s). (**c**) Carbon chains derived from monolayer graphene. Reprinted with permission from Ref. [83]. Copyright 2009, IOP Publishing Ltd. and Deutsche Physikalische Gesellschaft. (**d**) Black phosphorus nanoribbons with zigzag and armchair edges. Scale bar: 5 nm. Reprinted with permission from Ref. [84]. Copyright 2016, American Chemical Society.

A common strategy for the creation of 1D nanostructures is shown in Figure 6a. Firstly, the electron beam is focused to drill two nanopores. One nanopore expands under electron beam bombardment, and the bridge between the adjacent nanopore shrinks. Sustained electron beam irradiation increases the length and shrinks the diameter of the 1D structure.

Figure 6b provides an example of the fabrication of nanowires in a MoS_2_ monolayer, which confirms the feasibility of the above strategy [82]. The nanopores sculpted in MoS_2_ sheets with a focused beam at a current density of 40 A/cm^2^ grow to larger sizes under continuous 80 keV electron beam irradiation. As the nanopores expand, the region between adjacent pores narrows and ultimately transforms to a robust nonstoichiometric nanowire. Since S is more easily sputtered than Mo, a large number of Mo atoms are left at the edge of the nanopore and finally transform to a Mo-rich 1D structure. Such a top-down fabrication can produce ultrathin ribbons [26,85,86] and even atomic chains [87,88]. For instance, single-carbon chains have been fabricated in graphene membranes under 80 keV electron beam irradiation [83]. Figure 6c displays the evolution from a subnanometer nanoribbon to single-atom chains in suspended graphene. Accompanied by the removal of carbon atoms, the reconstruction of the ribbon involves more pentagons and heptagons than hexagons, which finally transform into single-carbon atom chain bridges between graphene contacts. In addition, atomic chains can also be fabricated in some other 2D materials, such as BN [85] and black phosphorus (BP) [86]. Apart from the size of the nanostructure being significantly controlled, various structures at the edge of nanoribbons can be sculpted along specific crystal directions. Taking BP as an example, BP ribbons with armchairs and zigzag edges can be obtained by changing the moving trajectory of the focused electron beam, as shown in Figure 6d [84].

### 4.3. 2D Structures

An energetic electron beam can promote the diffusion, aggregation, and rearrangement of atoms, which may facilitate the formation of 2D structures in a controllable way.

**Figure 7:**
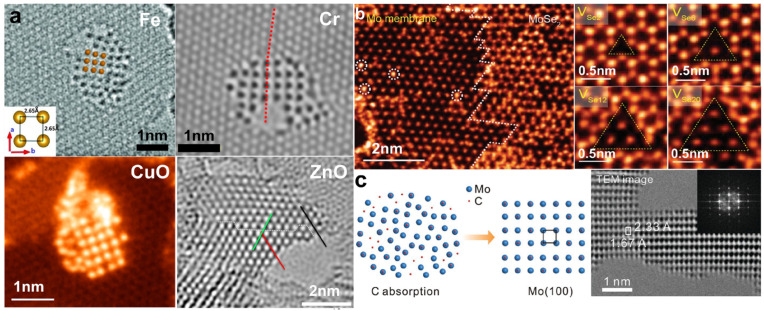
2D nanostructures formed under electron beam irradiation. (**a**) 2D membrane embedded in graphene holes. Reprinted with permission from Ref. [89]. Copyright 2014, American Association for the Advancement of Science. Reprinted with permission from Ref. [90]. Copyright 2020, American Chemical Society. Reprinted with permission from Ref. [94]. Copyright 2016, IOP Publishing Ltd. Reprinted with permission from Ref. [91]. Copyright 2015, American Chemical Society. (**b**) Mo membrane derived from a MoSe_2_ monolayer. Reprinted with permission from Ref. [95]. Copyright 2018, WILEY-VCH Verlag GmbH & Co. KGaA, Weinheim. (**c**) Ultrathin Mo membrane with a rectangular arrangement. Reprinted with permission from Ref. [96]. Copyright 2020, WILEY-VCH Verlag GmbH & Co. KGaA, Weinheim.

Many efforts have been made to obtain unique (quasi) 2D structures. It has been demonstrated that nanopores in 2D sheets can serve as templates for the growth of suspended quasi-2D structures [89,90,91]. As shown in Figure 7a, Fe film is the first metal monolayer successfully produced in graphene nanopores [92]. Fe atoms mainly come from the FeCl_3_ residue used during the preparation of graphene, and an 80 keV electron beam is used to promote the movement and reconstruction of Fe atoms. It is found that the Fe film is robust and keeps stable for several minutes. In a similar manner, a single-atom-thick Cr membrane with antiferromagnetism is experimentally obtained by reducing CrO using an electron beam irradiation of 80 keV [92]. Furthermore, it is predicted that dozens of metals can form 2D counterparts [93].

In addition to metal membranes, monolayer metal oxides can also be created in nanopores. As shown in Figure 7a, a CuO film with a square lattice is formed in graphene pores [94]. Under 60 keV irradiation, copper-oxide clusters and individual Cu atoms undergo movement, arrangement, and crystallization around the nanopore edge. Another example is the fabrication of mono- and bilayer ZnO coordinated in a trigonal-planar configuration in graphene nanopores [91]. In general, such nanopore-based fabrication is carried out under parallel electron beam irradiation with relatively low beam energy.

However, the geometry and size of 2D counterparts embedded in nanopores are constrained. To diminish the influence of the template, a novel strategy was proposed for the fabrication of Mo membranes from monolayer MoSe_2_ using a STEM [95]. As shown in Figure 7b, 80 keV electron beams are used to sputter Se atoms selectively in MoSe_2_ and Mo membranes embedded in the MoSe_2_ monolayer with clear boundaries forming at the regions where Se vacancies accumulated. The as-created Mo films are remarkably stable, and such a top-down approach can be applied to fabricate other metal membranes.

Another example is the fabrication of a symmetrical Mo membrane from 2D α-molybdenum carbide (α-Mo_2_C) using 80 keV electron beam irradiation [96], as shown in Figure 7c. C atoms are expected to be sputtered first due to their significantly higher sputtering cross-section, leaving Mo atoms rearranged into Mo membranes. It is noted that the structure of Mo films is highly influenced by foreign adatoms.

## 5. Automated Processing in 2D Materials at the Atomic Level

Despite many efforts, atomic fabrication at times cannot meet its promise in efficiency and consistency [97]. To resolve the issue, automation has been introduced into the atomic fabrication workflow to improve the control of the electron beam. Meanwhile, deep learning methods can be applied to analyze the (S)TEM images to extract structural information effectively for the adjustment of fabrication strategies and parameters. The electron beam can be focused onto an atom-size probe in the STEM, which has the potential to manipulate individual atoms and trigger structural evolution at the atomic scale [98,99,100,101]. Thus, automatic control systems incorporating STEMs are being used to explore atomic fabrication.

**Figure 8:**
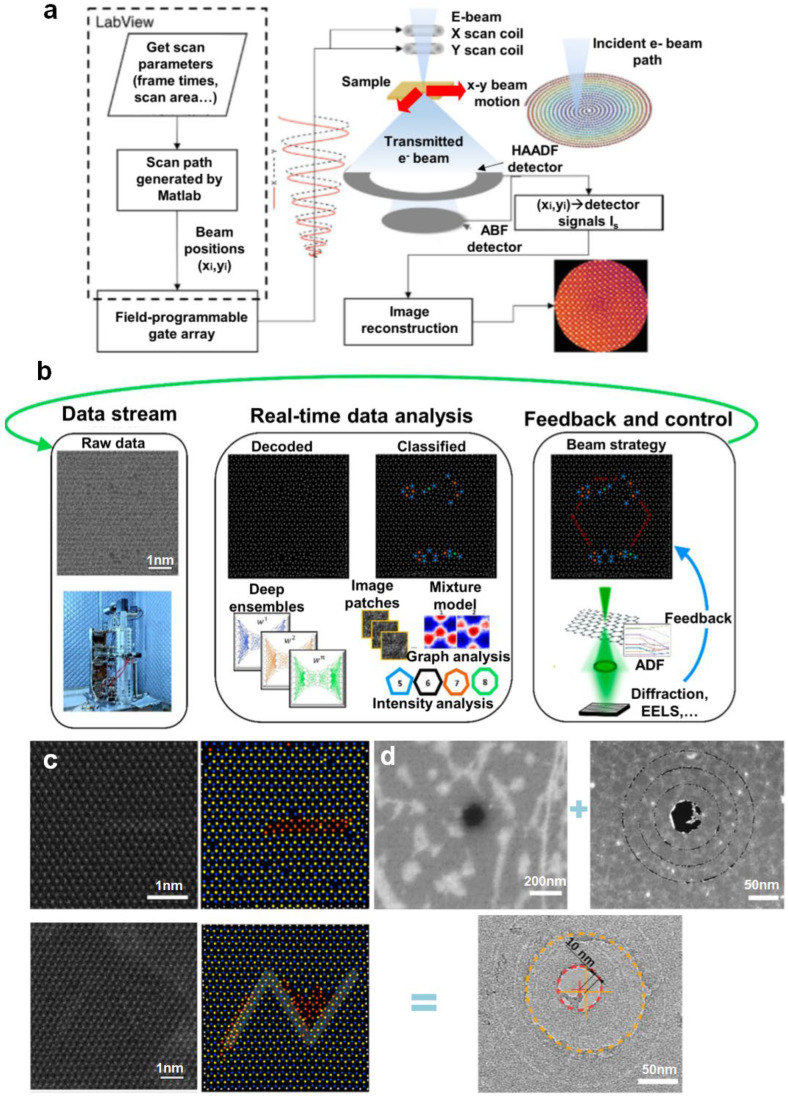
Schematics of automated fabrication. (**a**) Automated fabrication system based on a STEM. Reprinted with permission from Ref. [102]. Copyright 2017, the author(s). (**b**) Framework for atomic processing based on deep learning. Reprinted with permission from Ref. [103]. Copyright 2022, American Chemical Society. (**c**) Patterns formed by placing a single vacancy line in a specific location. Scale bar: 1 nm. Reprinted with permission from Ref. [103]. Copyright 2022, American Chemical Society. (**d**) Assembly of patterned graphene and a MoS_2_ membrane.Reprinted with permission from Ref. [105]. Copyright 2022, American Chemical Society.

Figure 8a is a schematic of an automatic system based on a STEM, which uses a field-programmable gate array (FPGA) to control the scan of an electron probe. Matlab codes provide input coordinates to the FPGA, and the electron beam in the STEM can move along a predefined path. Finally, output images are obtained by post-processing the signals from different detectors. As the maximum readout frequency is 2MHz, the FPGA system enables the microscope to scan with a high temporal resolution and produce images with an atomic-level resolution [102].

Given the complex manipulation and huge stream involved in automated processing, deep learning methods can be extended to electron beam manipulation and manufacturing feedback. Figure 8b presents a manipulation workflow based on a deep learning approach designed to fabricate single vacancy lines (SVLs) in MoS_2_ [103]. This system positions the electron beam to a specific coordinate after capturing a sample image. Only when the feedback results reach the setting threshold does the electron beam move to the next step. As shown in Figure 8c, target defects are placed as SVLs to form geometric patterns in MoS_2_. Based on the previous atomic identification and classification training, deep learning was used to identify the S_2_ site, and single sulfur atoms can be accurately removed at 60 kV voltage.

Another similar feedback-controlled system was reported by Boebinger et al. [104], using a focused electron beam with a sub-Å size to drill different patterns (circular, square, and triangular) in MoS_2_. The electron beam can scan along specific crystal directions. Finally, the nanopores obtained by triangular scanning in the zigzag sulfur-terminated direction are found to be the most stable, which is related to the lower values of the formation energy.

After nanopatterning 2D materials in a STEM, Haas et al. extended the atomic fabrication to 3D devices by combining a precise stacking operation. To obtain stacked structures, the Van der Waals force is employed to assemble monolayer 2D materials, such as graphene and BN [105]. This stacking structure can be characterized by the combination of patterns, as shown in Figure 8d, where the layer-by-layer assembly of graphene and MoS_2_ generates a group of concentric circles. This approach makes it possible to directly generate functional devices in 2D materials.

Currently, most automation in (S)TEMs mainly focuses on controlling the electron beam and its associated parameters, as well as using deep learning techniques to process the resulting images. However, there is still significant potential for advancing automation in other areas, such as specimen search, sample drift correction, and image capture. These aspects are also essential for achieving fully automated atomic manufacturing systems.

## 6. Conclusions and Outlook

The manipulation and fabrication of 2D materials is crucial for achieving superior properties and potential applications; (S)TEMs provide an available platform for atomic fabrication due to their capability to trigger and monitor structural evolution at the atomic scale using an electron beam. The incorporation of automated control systems may help to enable microscopes to tailor structures of materials with a high accuracy and consistency. This review summarizes the electron-beam-induced atomic dynamics and controllable fabrication of desired structures using an electron microscope. Despite the great progress already achieved, there are still many challenges and opportunities.

Firstly, atomic fabrication is still in its infancy, and the dynamics of atomic-scale evolution remain elusive. It is expected that structural evolution at the atomic scale is distinct from that at the microscale and macroscale; many phenomena are difficult to interpret and predict with traditional theory, introducing great uncertainty to the understanding of structural evolution and the development of new methods for atomic fabrication. Although in situ TEM studies have provided novel insights into the evolution dynamics, some important intermediate states and products during fabrication may be missed because conventional instruments are too slow to probe ultrafast structural dynamics. Therefore, time-resolved microscopy should be further developed to acquire images with atomic resolution as fast as possible. Moreover, the foundation for the development of fabrication can expand to various signals collected simultaneously with electron beam processing, which will provide more comprehensive information to confirm the fabrication process and promote accuracy.

Secondly, atomic fabrication is still in its exploratory phase, and fabrication output is usually small-scale. There is a long way to go before atomic fabrication achieves industrialization. Some techniques, such as automation, have been pivotal in scaling up fabrication and improving efficiency. In the future, the integration of database indexing, atomic modeling, artificial intelligence, and other advanced algorithms is expected to drive significant improvements in efficiency, thereby unlocking the potential for industrialization. Meanwhile, parallel processing approaches, such as multi-probe processing, are expected to be promising solutions for achieving scalability.

Lastly, most of the investigations of atomic fabrication are carried out only under electron beam irradiation using (S)TEMs, which is expected to extend to a combination with external fields. However, only a small fraction of atomic fabrication has undergone detailed mechanistic examination using advanced in situ (S)TEMs because the setup of real fabrication conditions inside a microscope is difficult. The introduction of external fields or environments by dedicated holders enables the microscope to dynamically observe the evolution dynamics and to further investigate the influence of the external fields and environments on the fabrication. It is noted that observed structural evolution actually stems from a combined effect of the applied fields and the electron beam irradiation. Decoupling these effects and confirming the influence of each stimulus requires further detailed study. On this foundation, (S)TEMs equipped with dedicated holders will play a significant role in the further investigation of atomic fabrication.

## Data Availability

Not applicable.
